# *Mycobacterium tuberculosis nuoG* Is a Virulence Gene That Inhibits Apoptosis of Infected Host Cells

**DOI:** 10.1371/journal.ppat.0030110

**Published:** 2007-07-20

**Authors:** Kamalakannan Velmurugan, Bing Chen, Jessica L Miller, Sharon Azogue, Serdar Gurses, Tsungda Hsu, Michael Glickman, William R Jacobs, Steven A Porcelli, Volker Briken

**Affiliations:** 1 Department of Cell Biology and Molecular Genetics, University of Maryland, College Park, Maryland, United States of America; 2 Howard Hughes Medical Institute, Albert Einstein College of Medicine, Bronx, New York, United States of America; 3 Division of Infectious Diseases, Immunology Program, Memorial Sloan Kettering Cancer Center, New York, New York, United States of America; 4 Department of Microbiology and Immunology, Albert Einstein College of Medicine, Bronx, New York, United States of America; 5 Department of Medicine, Albert Einstein College of Medicine, Bronx, New York, United States of America; Johns Hopkins School of Medicine, United States of America

## Abstract

The survival and persistence of Mycobacterium tuberculosis depends on its capacity to manipulate multiple host defense pathways, including the ability to actively inhibit the death by apoptosis of infected host cells. The genetic basis for this anti-apoptotic activity and its implication for mycobacterial virulence have not been demonstrated or elucidated. Using a novel gain-of-function genetic screen, we demonstrated that inhibition of infection-induced apoptosis of macrophages is controlled by multiple genetic loci in M. tuberculosis. Characterization of one of these loci in detail revealed that the anti-apoptosis activity was attributable to the type I NADH-dehydrogenase of *M. tuberculosis,* and was mainly due to the subunit of this multicomponent complex encoded by the *nuoG* gene. Expression of *M. tuberculosis nuoG* in nonpathogenic mycobacteria endowed them with the ability to inhibit apoptosis of infected human or mouse macrophages, and increased their virulence in a SCID mouse model. Conversely, deletion of *nuoG* in M. tuberculosis ablated its ability to inhibit macrophage apoptosis and significantly reduced its virulence in mice. These results identify a key component of the genetic basis for an important virulence trait of M. tuberculosis and support a direct causal relationship between virulence of pathogenic mycobacteria and their ability to inhibit macrophage apoptosis.

## Author Summary

The infection-induced suicide of host cells following invasion by intracellular pathogens is an ancient defense mechanism observed in multicellular organisms of both the animal and plant kingdoms. It is therefore not surprising that persistent pathogens of viral, bacterial, and protozoal origin have evolved to inhibit the induction of host cell death. *M. tuberculosis,* the etiological agent of tuberculosis, has latently infected about one third of the world's population and can persist for decades in the lungs of infected, asymptomatic individuals. In the present study we have identified *nuoG* of *M. tuberculosis,* which encodes a subunit of the type I NADH dehydrogenase complex, as a critical bacterial gene for inhibition of host cell death. A mutant of M. tuberculosis in which *nuoG* was deleted triggered a marked increase in apoptosis by infected macrophages, and subsequent analysis of this mutant in the mouse tuberculosis model provided direct evidence for a causal link between the capacity to inhibit apoptosis and bacterial virulence. The discovery of anti-apoptosis genes in M. tuberculosis could provide a powerful approach to the generation of better attenuated vaccine strains, and may also identify a new group of drug targets for improved chemotherapy.

## Introduction

Tuberculosis is an infectious disease of enormous and increasing global importance. Currently, about one third of all humans are latently infected with its etiologic agent, Mycobacterium tuberculosis (Mtb), and an estimated 2.5 million people die of tuberculosis annually [1]. After infection of a mammalian host, Mtb is able to resist innate host defenses sufficiently to increase the local bacterial burden and disseminate throughout the body. With the onset of the adaptive immune response, however, the bacterial numbers are controlled in over 90% of infected individuals. Nevertheless, the host is not able to completely clear the bacterial burden, thus leading to persistence of Mtb within the lungs and other tissues of healthy individuals. These latent infections can be reactivated to generate full-blown disease, a process that is accelerated by immunocompromised states resulting from senescence, malnutrition, and co-infection with HIV, which is a major source of morbidity and mortality associated with the current HIV epidemics in many countries [2–5].

Programmed cell death (apoptosis) plays an important role in the innate immune response against pathogens and comprises an evolutionarily conserved defense strategy that extends even into the plant world [6,7]. It is therefore essential for persisting intracellular pathogens to have strong anti-apoptosis mechanisms [8–12]. While a few studies have suggested that under some conditions Mtb may induce host cell apoptosis [13–16], a substantial body of evidence points strongly to the expression of strong anti-apoptotic mechanisms by Mtb and other closely related virulent bacteria. Furthermore, this capacity is not found in avirulent species, suggesting a causal link between virulence and inhibition of macrophage apoptosis [17–19]. This hypothesis is supported by the recent discovery that the genetic predisposition of different inbred mouse strains to mycobacterial infections is linked to the capacity of their macrophages to undergo apoptosis or necrosis upon infection, with the former response imparting a resistant and the latter a susceptible host phenotype [20].

Further confirmation of the findings that Mtb inhibits host cell apoptosis is provided by a number of studies that have addressed its molecular mechanism. The importance of Mtb-induced upregulation of anti-apoptosis genes in infected macrophages for apoptosis inhibition was supported by functional data using either anti-sense oligonucleotides to knock down mcl-1 expression [19] or A1 knock-out mice lacking the anti-apoptosis gene A1 [21,22]. These results implicate the intrinsic (mitochondria-mediated) apoptosis pathway as a target for Mtb-mediated apoptosis inhibition, because mcl-1 and A1 are both members of the large family of Bcl-2–like proteins that localize prominently to mitochondria. However, this is contradicted by the finding that overexpression of Bcl-2 (another mitochondrial anti-apoptotic protein) could not rescue cells from undergoing apoptosis after infection with nonvirulent mycobacteria, thus suggesting that the extrinsic pathway (death receptor–mediated) is involved in the infection-induced apoptosis [23]. Consistently, virulent Mtb strains could inhibit FasL-induced apoptosis in Fas-expressing cells [18]. The same group reported very recently that lipoglycans of the Mtb cell wall stimulate the activation of NF-kB via TLR-2 and that the subsequent upregulation of cellular FLIP leads to inhibition of FasL-mediated apoptosis [24]. Furthermore, it was suggested that Mtb stimulated the secretion of soluble TNF-R2, which led to the reduction of bioactive TNF-α in the medium and therefore less stimulation of the TNF-R1 [25]. Altogether, it seems that virulent Mtb is able to inhibit induction of host cell apoptosis via multiple pathways, and probably encodes mechanisms to interfere with both intrinsic and extrinsic pathways for initiation of programmed cell death.

The inhibition of macrophage apoptosis by Mtb is believed to provide a number of advantages to the bacterium in its struggle to resist the host immune response. These include preservation of a favorable host cell environment during growth and persistence [26,27], evasion of apoptosis-linked bactericidal effects [28,29], and avoidance of efficient cytotoxic T cell priming via the detour pathway of antigen cross-presentation [15,30–32]. This last point is of potential importance to the improvement of tuberculosis vaccines, because attenuated mycobacterial strains that induce higher levels of host cell apoptosis would be expected to stimulate more robust cellular immunity, as suggested by a recent study using recombinant M. bovis Bacille Calmette-Guérin (BCG) expressing listeriolysin [33,34]. Therefore, the identification of mycobacterial genes required for prevention of apoptosis could lead to specific strategies for designing more efficacious forms of BCG or other attenuated mycobacterial vaccine strains.

In order to clarify the role of mycobacteria in host cell apoptosis and to address its importance for bacterial virulence, we sought to identify anti-apoptosis genes via a gain-of-function genetic screen. Using this approach, we successfully identified two independent genomic regions of virulent Mtb (strain H37Rv) that mediate the inhibition of host cell apoptosis by the facultative pathogen *M. kansasii*. The analysis of a defined set of bacterial mutants of *M. kansasii* in immunocompromised (SCID) mice demonstrated a causal relationship between inhibition of apoptosis and virulence. These findings were confirmed via a loss-of-function strategy using the newly identified anti-apoptosis gene, *nuoG,* and demonstrated attenuation of Mtb *nuoG* mutants in immunocompromised and immunocompetent mice. Altogether our findings allowed, to our knowledge for the first time, the demonstration of a causal relationship between inhibition of host cell apoptosis and virulence of mycobacteria.

## Results/Discussion

### Gain-of-Function Genetic Screen for Anti-Apoptosis Genes in Mtb

**Figure 1:**
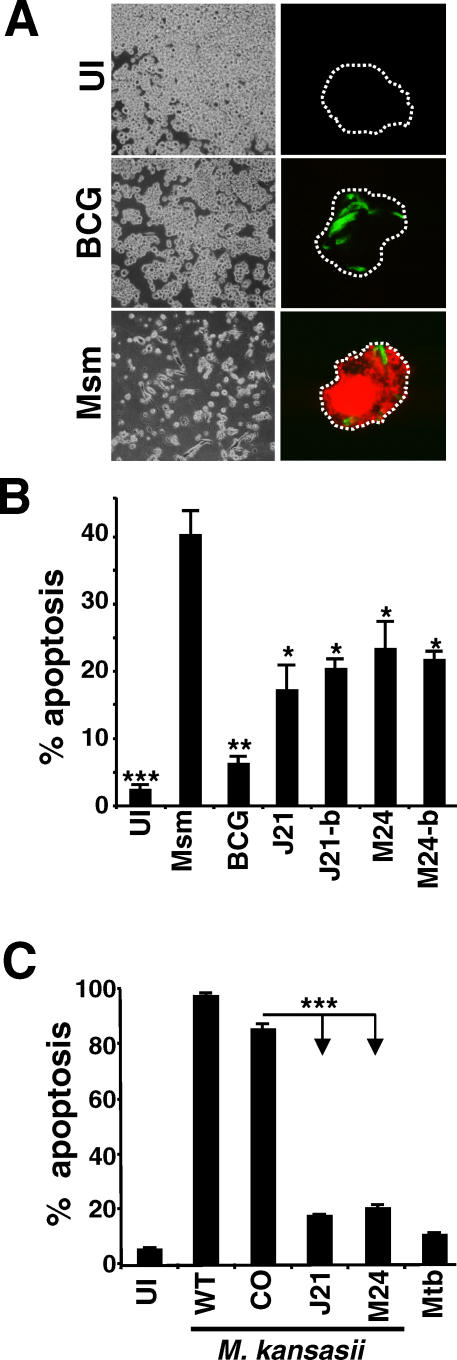
Identification of Regions in the Mtb Genome Mediating Inhibition of THP-1 Cell Apoptosis (A) M. smegmatis (Msm) induced more cell death in infected THP-1 cells than BCG or uninfected cells (UI) as observed by bright field microscopy (left panels) and fluorescence microscopy of TUNEL staining (right panels; red fluorescence is TUNEL staining, and green fluorescence is GFP-labeled bacteria). (B and C) *M. smegmatis* was transfected with an episomal cosmid library of Mtb genomic DNA, and individual clones were screened for their capacity to inhibit apoptosis. The cosmid DNA of two selected clones (J21 and M24) was purified and used to re-transfect *M. smegmatis,* resulting in clones J21-b and M24-b. These clones, along with the original transformants (M24 and J21), uninfected (UI) cells, M. smegmatis (Msm), and BCG were tested for induction of apoptosis by infection of THP-1 cells followed by TUNEL staining at 16 h after infection and flow cytometry (C). The J21 and M24 cosmids and empty vector cosmid (CO) were transfected into *M. kansasii,* and the induction of apoptosis by the bacteria was compared to uninfected and Mtb-infected THP-1 cells using a TUNEL assay as in (B), except that cells were harvested after 5 d of infection. Results in (B and C) are averages of three independent experiments and error bars represent ± standard deviation (SD). Statistical significance relative to levels of apoptosis induced by wild-type M. smegmatis in (B) or M. kansasii in (C) is indicated as follows: *, 0.01 < *p* < 0.05; **, 0.001 < *p* < 0.01; ***, *p* < 0.001 (ANOVA with Tukey post-test).

To identify genes in Mtb responsible for anti-apoptotic effects, we established a gain-of-function genetic screen using the nonpathogenic M. smegmatis mc^2^155 strain, which is a fast-growing mycobacterium that is extremely efficient for transformation [35]. The human cell line THP-1 was chosen for use as the host cells for this screen because published work indicates that these cells provide an accurate model for the apoptotic response of Mtb-infected primary human alveolar macrophages [23]. Our initial studies established that M. smegmatis infection of THP-1 cells induced strong apoptosis after 1.5 d of infection when compared to BCG-infected macrophages, as assessed by disruption of the cell monolayer and by staining for DNA strand breakage using the TUNEL assay (Figure 1A). The failure of BCG to induce apoptosis seems to contradict the report by Kean et al. [17] in which seven different species of mycobacteria were compared for their capacity to induce apoptosis in primary human alveolar macrophages, and BCG was among the apoptosis-inducing mycobacterial species. Nevertheless, in that study, no fast-growing species were included, and all apoptosis assays were performed after 5–7 d of infection. In an independent study, we demonstrated that M. smegmatis and other nonpathogenic mycobacteria like M. fortuitum have a very strong capacity to induce apoptosis with rapid kinetics, even when compared to facultative pathogenic mycobacteria like M. kansasii and BCG (A. Bohsali and V. Briken, unpublished data). Therefore, the induction of apoptosis by M. smegmatis had to be analyzed very early after infection (16–36 h; Figure 1A and 1B), and at this time point, little or no induction of macrophage apoptosis by BCG could be observed.

The gain-of-function screen was performed using a library of 312 M. smegmatis clones containing Mtb genomic DNA fragments on an episomal cosmid. Two clones (designated J21 and M24) containing cosmids with separate nonoverlapping Mtb genomic DNA inserts gave significantly reduced levels of apoptosis and were selected for detailed study. To confirm that the observed effects on apoptosis were due to the cosmids contained in these clones, the episomal cosmid DNA was extracted and re-transfected into M. smegmatis. In both cases, the re-transformed clones had the same phenotype as the original clones, showing an approximately 50% reduction of apoptosis of infected THP-1 cells (Figure 1B). It is important to note that our screening was designed to emphasize specificity rather than sensitivity, as it focused on the clones showing the strongest and most reproducible suppression of apoptosis. Thus, it is very likely that other regions with anti-apoptosis capacity may remain to be identified.

Although the effects of cosmids M24 and J21 on reducing the apoptosis induced by M. smegmatis were highly reproducible, the magnitude of this effect was relatively modest. Most likely, this reflected the very strong capacity of M. smegmatis to induce apoptosis when compared to other mycobacteria (Figure 1 and unpublished data), and we assessed this by testing the effects of the Mtb cosmids on apoptosis induction by another mycobacterial species, M. kansasii. This opportunistic pathogen is known to be a strong inducer of apoptosis, but it shows slower host cell killing than *M. smegmatis,* with significant levels of apoptosis being observed only after 5–7 d of infection [17]. We transformed the cosmids into M. kansasii to generate clones Mkan-J21 and Mkan-M24, and also generated an M. kansasii control strain using the empty cosmid vector pYUB415 (Mkan-CO). Based on FACS analysis of TUNEL staining, wild-type M. kansasii and Mkan-CO induced comparable high levels of apoptosis at 5 d post infection (95% and 86% apoptosis, respectively), whereas Mkan-J21 and Mkan-M24 showed markedly reduced levels (16% and 19% apoptosis, respectively) (Figure 1C). The transfection of the two cosmid clones did not affect the in vitro growth of either M. smegmatis or M. kansasii (unpublished data).

### Inhibition of Apoptosis Caused an Increase in Virulence of Nonpathogenic *M. kansasii*

**Figure 2:**
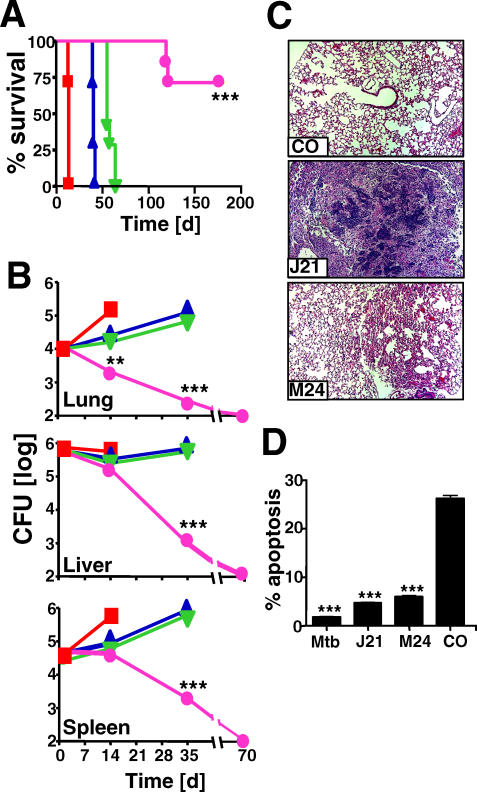
Correlation between Apoptosis Inhibition and Mycobacterial Virulence (A and B) SCID mice were infected intravenously with 10^6^ bacteria and survival ([A], *n* = 7 per group), or the bacterial load in lung, liver, and spleen ([B], *n* = 3 per time point), were determined. Shown are results with Mtb (squares), Mkan-J21 (triangle), Mkan-M24 (inverted triangle), and Mkan-CO (circle). (C) Histopathology (hematoxylin and eosin staining) of lungs from SCID mice infected 5 wk earlier with Mkan-CO, Mkan-J21, or Mkan-M24 as indicated. (D) Levels of apoptosis in vivo after 2 wk of infection were determined by quantification of TUNEL-positive cells per total cell nuclei in lung tissue sections. Means ±SD for blinded analysis of about 2,000 total cells per tissue section (three sections per lung) per mouse (*n* = 3) are shown. The results shown are representative of three independent experiments. Error bars representing ±SD are shown in (D) and are smaller than symbols in (B). Asterisks indicate *p*-values as in Figure 1.

Apoptosis of infected macrophages has been reported to directly kill ingested bacteria [29], and killing of bacteria within apoptotic bodies is also facilitated as a result of enhanced phagocytosis by activated bystander macrophages [28]. Therefore, we hypothesized that inhibition of apoptosis is important for mycobacterial evasion of the host's innate immune response. This was tested by infecting groups of SCID mice (BALB/c background) with the M. kansasii cosmid transformants or with Mtb H37Rv. As expected, H37Rv was highly virulent in these mice that lack adaptive immunity (median survival 15 d), whereas Mkan-CO showed only modest virulence even in these immunodeficient animals (median survival >200 d) (Figure 2A). Remarkably, Mkan-J21 and Mkan-M24 showed significantly increased virulence in SCID mice, with median survival times of 44 d or 60 d, respectively (*p* = 0.0002 compared to Mkan-CO for both survival times by log-rank test). Tissue bacterial burdens in SCID mice infected with the various mycobacteria were consistent with the survival data, based on colony-forming units (CFUs) in the lung, liver, and spleen at various time points after infection (Figure 2B). Histopathology of the lungs revealed that after 35 d, airways in the lungs of Mkan-J21–infected mice were almost completely consolidated, and in Mkan-M24–infected mice the lungs showed significant infiltration of inflammatory cells. In contrast, lungs of mice infected with Mkan-CO had normal morphology (Figure 2C). In order to correlate the virulence of the different strains with their capacity to inhibit apoptosis, lung sections obtained 14 d after infection were stained for apoptotic cells using a TUNEL-based assay and analyzed by microscopy as described in the Materials and Methods section. This revealed minimal levels of apoptosis in situ for lung tissue infected with H37Rv (2%), Mkan-J21 (5%), and Mkan-M24 (7%) compared to significantly higher levels in Mkan-CO–infected (27%) lungs (Figures 2D and S1). Overall, these results confirmed the anti-apoptotic activity of genes contained in cosmids J21 and M24, and strongly supported the importance of apoptosis inhibition for the virulence of mycobacteria.

### Identification of *nuoG* as an Anti-Apoptosis Gene of Mtb

Cosmids J21 and M24 both contained about 30 mycobacterial genes, and therefore it could not be completely excluded that the effect on bacterial virulence was in part or totally caused by another gene or genes linked to those responsible for the anti-apoptosis effects. As Mkan-J21 showed the strongest enhancement of virulence in SCID mice, we selected cosmid J21 for additional studies to determine the precise gene or genes required for inhibition of apoptosis by M. tuberculosis. Sequencing of J21 cosmid DNA (unpublished data) showed that its insert corresponded to bp 3511794 through bp 3545572 of the Mtb genome, according to the standard annotation for strain H37Rv [36]. This interval contains the intact open reading frames of 31 annotated genes, including a large operon that encodes the 14 subunits of the Mtb type I NADH dehydrogenase complex (NDH-1), along with other genes that encode a variety of different known or predicted functions (Figure S2).

To identify which gene or genes in this region were important for the anti-apoptosis activity, a series of deletion mutants spanning different regions within the genomic interval corresponding to J21 was created in Mtb H37Rv using specialized transduction [37] (Figures S2 and S3). These mutants were analyzed by using SCID mice to test for reduction in bacterial virulence (Figure S4A), and by using THP-1 cells to test for loss of apoptosis inhibition (Figure S4B). Although several of the deletions showed modest effects, only one mutant, Mtb Δ*Rv3151,* which corresponded to the deletion of the *nuoG* subunit of NDH-1, gave both statistically significant extension of survival in SCID mice and enhanced apoptosis in THP-1 cells relative to parental Mtb. The gain-of-function experiments described in Figures 1 and 2 using the episomal cosmids relied on the proper function of the endogenous gene promoters in transfected M. smegmatis or M. kansasii. To confirm that *nuoG* was actually transcribed on the J21 cosmid in *M. smegmatis,* reverse transcription–PCR with primers specific for Mtb nuoG was performed and clearly demonstrated that the nuoG gene was transcribed (Figure S4C). These experiments implicated *nuoG,* and potentially the complete functional NDH-1 complex, in mediating most or all of the anti-apoptotic properties of cosmid clone J21.

**Figure 3:**
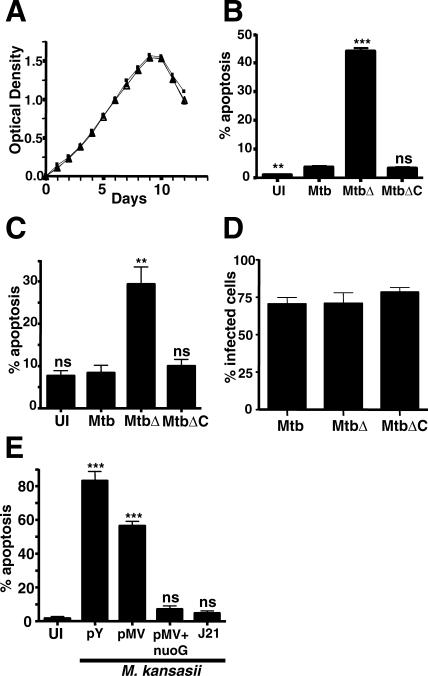
Importance of Mtb *nuoG* for Inhibition of Macrophage Apoptosis (A) The in vitro growth of wild-type (squares), mutant (full triangles), and complemented bacteria (empty triangles) was analyzed at the indicated time points in triplicate cultures (error bars showing ±SD are smaller than symbols). (B and C) Induction of apoptosis by infection of differentiated human THP-1 cells (B) or cultured primary mouse macrophages (C) with wild-type (Mtb), the *ΔnuoG* deletion mutant (MtbΔ), or complemented mutant (MtbΔC). Cells were harvested either at day 3 (B) or day 1 (C) after infection, stained by TUNEL assay, and analyzed using flow cytometry. (D) The percentage of infected macrophages in (C) was determined by acid-fast staining. (E) M. kansasii was transfected with empty cosmid (pY), empty plasmid (pMV), cosmid J21 (J21), and Mtb *nuoG* behind the constitutive promoter of pMV261 (pMV+nuoG), and induction of apoptosis in THP-1 cells was determined by TUNEL staining and flow cytometry. All results shown are means ±SD of triplicate cultures and are representative of three independent experiments. Asterisks indicate statistical significance as in Figure 1. ns, not significant.

**Figure 4:**
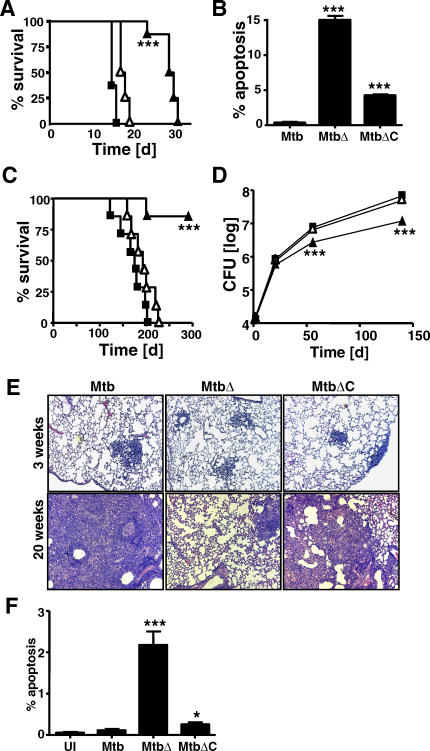
Identification of *nuoG* as a Mycobacterial Virulence Determinant (A) Survival of SCID mice after intravenous infection with 10^6^ wild-type Mtb (squares), Δ*nuoG* mutant (filled triangles), or complemented mutant bacilli (open triangles). *n* = 7 mice per group. (B) Apoptotic cells in lung tissues of SCID mice after 14 d of infection were quantified using TUNEL peroxidase staining by microscopy as explained in Figure 2. (C) Survival of immunocompetent BALB/c mice infected with bacterial strains as in (A); *n* = 7 mice per group. (D) The bacterial burden in the lungs of infected BALB/c mice was followed (*n* = 3 per time point; symbols indicate bacterial strains as in [A and C]). (E) Lung histopathology (hematoxylin and eosin staining) at 3 and 20 wk for BALB/c mice infected as in (C) with wild-type Mtb, Δ*nuoG* mutant (MtbΔ), or complemented mutant Mtb (MtbΔC). (F) The percentages of apoptotic cells in lung sections were determined by TUNEL peroxidase assay and quantified in blinded fashion as in Figure 2. All results shown are representative of two independent experiments. Statistically significant differences compared to wild-type Mtb are indicated by asterisks as in the Figure 1 legend.

To confirm that *nuoG* deletion was responsible for the pro-apoptotic phenotype and to exclude significant polar effects of the deletion on other nearby genes, the mutant was complemented with a plasmid carrying a copy of *nuoG* behind a constitutively active promoter that integrates into the Mtb chromosome at the unique *attB* site [38]. This gave full complementation for the in vitro apoptosis assays (Figure 3), although there was a residual increase in apoptosis induction observed in vivo (Figure 4B and 4F) that may have been due to some minor polar effects on the transcription of other members of the nuo-operon. The mutant (MtbΔ), complemented mutant (MtbΔC), and wild-type Mtb were analyzed for the capacity to grow in vitro, which demonstrated that the *nuoG* deletion had no effect on aerobic growth rate (Figure 3A), confirming a previous report that NDH-1 is not essential for mycobacterial growth in culture [39]. This is most likely due to the fact that Mtb has two additional NADH dehydrogenases, which are the non-proton pumping type II NADH dehydrogenases encoded by the *ndh* and *ndhA* genes [40].

To confirm the pro-apoptotic effect of *nuoG* deletion, we compared levels of apoptotic cell death in cultures of differentiated THP-1 cells and extended the analysis to include detection of apoptosis in bone marrow–derived macrophages (BMDMs) from BALB/c mice following infection. The Mtb *nuoG* mutant induced significantly more apoptosis than the complemented strain or the wild-type Mtb in human and mouse macrophages (Figure 3B and 3C). This phenotype of the *nuoG* mutant was also confirmed in BMDMs from C57BL/6 mice (unpublished data). The difference in apoptosis induction was not due to a reduced phagocytosis of the *nuoG* mutant, which was demonstrated by comparing rates of infection via acid-fast staining (Figure 3D) and CFU determination (unpublished data). These results show that *nuoG* was necessary for Mtb to inhibit apoptosis of primary murine macrophages or the human macrophage-like THP-1 cells. The *nuoG* Mtb mutant still induced less apoptosis (Figure 3B) when compared to *M. kansasii*–infected cells (Figure 1C), which probably reflects the fact that virulent Mtb expresses multiple anti-apoptosis genes. Therefore, the deletion of only one gene does not completely abolish the capacity of the bacterium to inhibit apoptosis. Since nuoG is part of a multi-subunit NDH-1 complex that is present in the cosmid J21, it was of interest to determine if nuoG alone could confer the gain-of-function in *M. kansasii* or if, in contrast, expression of the whole nuo-operon was needed. Therefore, Mtb-nuoG was constitutively expressed in *M. kansasii* and the capacity to induce host cell apoptosis was analyzed (Figure 3E). This demonstrated that expression of Mtb-nuoG alone very significantly reduced apoptosis induction by *M. kansasii* when compared to that of wild-type and empty vector-transfected bacteria (Figure 3E).

Most of the sequenced genomes of mycobacteria contain a *nuoG* gene within a nuo-operon containing 14 genes that code for the NDH-1. The one exception to date is *M. leprae,* in which the whole operon is deleted except for a *nuoN* pseudogene [41]. *nuoG* of M. kansasii was cloned using PCR and sequenced in order to allow protein sequence comparison of all the nuoGs in the mycobacterial strains used in our study. Comparison of the nuoG protein sequences among virulent mycobacteria revealed a high degree of homology (99% identity, Figure S9). Interestingly, nuoG of BCG is also highly homologous (99%) to nuoG of virulent mycobacterial species, which is consistent with our unpublished data demonstrating that the deletion of *nuoG* in BCG also increases the potential of the bacteria to induce apoptosis. These findings suggest that the vaccine strain BCG retained this virulence mechanism from its parental M. bovis strain, although overall it may still induce more apoptosis than fully virulent mycobacteria. In contrast, nuoG of M. smegmatis is only 70% identical (Figure S9) and nuoG of M. kansasii is only 34% identical to nuoG sequences of virulent mycobacterial species. The latter nuoG protein is truncated due to a stop codon introduced at codon 295, and thus nuoG of M. kansasii is missing about 512 of the C-terminal amino acids. Interestingly, the M. kansasii nuoG is quite homologous up to amino acid 288 (94%). In conclusion, it seems likely that both nuoG of M. smegmatis and M. kansasii have lost their apoptosis-inhibiting function within the NDH-1 complex, and therefore the overexpression of Mtb nuoG is able to restore the capacity of the bacteria to inhibit host cell apoptosis. It will be of great interest to explore this hypothesis further by examining the expression of the various nuoG proteins in the nuoG deletion mutant of Mtb and analyzing the apoptosis induction of these complemented bacteria.

How does nuoG mediate apoptosis inhibition? For nuoG to have a direct effect on host cell apoptosis pathways, one would assume that it needs to be secreted in order to interact with host cell proteins or lipids. However, if the structure of the NDH-1 complex of Mtb is similar to that of other bacterial NDH-1 complexes, nuoG will be located in the cytosol of the bacterium [42]. To determine whether Mtb nuoG is secreted experimentally, we created a phoA-nuoG fusion protein. phoA can convert a colorless substrate into a blue product, but only if it is secreted by the bacterium [43]. This assay failed to detect secretion of nuoG (Figure S7), which is consistent with the absence of a signal peptide and the predicted cytosolic localization of this component of NDH-1. Altogether, it thus seems unlikely that nuoG is secreted, and a direct effect of nuoG onto the host cell can also be judged to be unlikely.

The disruption of the NDH-1 system in the nuoG mutant might have a very profound impact on the metabolism and proteome of the mycobacterium, which might result in an indirect effect on host cell apoptosis induction. Nevertheless, the absence of an in vitro growth defect in the nuoG mutant (Figure 3A) would argue against a profound effect of the deletion on bacterial metabolism. In order to address the effect of the nuoG mutation on the proteome of the mycobacteria, the proteins of wild-type and mutant Mtb were separated via 2-D gel electrophoresis. This revealed that the nuoG mutation did not induce a major change in the proteome (Figure S8). Thus, we found that the deletion of nuoG did not have a major impact on the general bacterial metabolism or its proteome. Instead, we propose that nuoG exerts its anti-apoptotic and virulence-promoting function via the enzymatic activity of the NDH-1 complex in a more specific way.

### Importance of Inhibition of Host Cell Apoptosis for Virulence of Mtb

Regardless of the remaining questions about the potential mechanism of the nuoG/NDH-1–mediated apoptosis inhibition, our identification of an apoptosis-inducing mutant of Mtb allowed us to analyze the importance of apoptosis inhibition for bacterial virulence. First, the importance of host cell apoptosis inhibition in innate immune defense was analyzed by infecting immunodeficient SCID mice. The median survival times for mice infected with Mtb wild-type or the complemented strain were not significantly different (14 d and 16.5 d, respectively). In contrast, mice infected with Δ*nuoG* Mtb survived twice as long as those infected with wild-type bacteria (median survival of 27.5 d, *p* < 0.0001, log-rank test; Figure 4A), even though all mice received similar initial bacterial doses as confirmed by CFU counts at day 1 after infection (Figure S10). Consistently, the amount of apoptosis induced in lung sections of these mice was significantly increased from less than 1% with wild-type bacteria to about 13% in mutant bacteria (*p* < 0.05) (Figures 4B and S5). The complemented bacteria still showed increased apoptosis (6%), but this was significantly reduced compared to the *nuoG* mutant (*p* < 0.05). Therefore, the deletion of *nuoG* significantly increased the induction of apoptosis in the lungs of SCID mice. These results corroborated the findings presented in Figure 2 by using a loss-of-function approach, and together both sets of experiments point towards an important role of infection-induced apoptosis in the innate immune response. This statement is supported by the recent results linking the capacity of host cell macrophages to undergo apoptosis upon mycobacterial infection to the susceptibility of different mouse strains [20].

The importance of nuoG-mediated apoptosis inhibition for bacterial virulence in immunocompetent mice was analyzed using BALB/c mice (Figure 4C–4F). Again, the mutant significantly (*p* < 0.004) delayed the death of infected mice when compared to wild-type (median survival 175 d) and complemented bacteria (median survival 193 d, *p* = 0.16 compared to wild-type) (Figure 4C). Measurement of bacterial CFUs showed that the growth of the mutant in the lungs of BALB/c mice was significantly reduced by approximately 0.8 log at week 20, after similar initial growth during the first 3 wk of infection (Figure 4D). In contrast, the bacterial loads in spleen and liver were not significantly different at 3, 10, or 20 wk post infection (unpublished data). Comparison of the histopathology of lung sections of infected BALB/c mice demonstrated an obvious reduction in granulomatous inflammation at week 20 in animals infected with the Δ*nuoG* mutant (Figure 4E). Although lung histopathology appeared similar in wild-type and Δ*nuoG* mutant–infected BALB/c mice at 3 wk (Figure 4E), staining of lung sections at this time point for TUNEL reactivity revealed a significant increase of greater than 10-fold in apoptotic cells in mice infected with the Δ*nuoG* mutant compared to mice that were uninfected, or infected with wild-type or complemented bacteria (Figures 4F and S6).

Taken together, our results demonstrate that *nuoG* is an anti-apoptosis gene of Mtb that is important for bacterial virulence in both immunocompromised and immunocompetent mice and thus strongly support the general hypothesis that the inhibition of host cell apoptosis is important for virulence of mycobacteria. The challenge ahead is to determine the molecular mechanism by which a bacterial NADH dehydrogenase can manipulate host cell apoptosis induction. It is intriguing to speculate that perhaps the NDH-1 complex of virulent bacteria has taken on a separate function in modifying apoptotic responses of infected macrophages from its original purpose of energy generation, which is now mainly performed by the type II dehydrogenases *ndh* and *ndhA,* which are both essential genes. One of the unique features of NDH-1, as opposed to the NDH and NDHA dehydrogenases of *M. tuberculosis,* is its capacity to pump protons across the bacterial membrane. We therefore hypothesize that these protons, in conjunction with the secreted bacterial superoxide dismutases (SodA and SodC), could serve to neutralize the superoxide anions generated within the phagosome by an activated NOX2 complex to generate hydrogen peroxide, which is then further catabolized to water and oxygen by bacterial catalase (KatG). Superoxide anions are a known trigger for apoptosis in a variety of biological systems, so the involvement of NDH-1 and nuoG in their elimination may interrupt a critical signal that initiates the host cell apoptosis response. Our hypothesis would predict that other NDH-1 subunits involved in proton translocation, such as nuoL and nuoM [42], will have the same apoptosis phenotype as the nuoG mutant, a prediction that has not yet been tested experimentally. This proposed general mechanism for inhibition of apoptosis is further supported by other studies implicating SodA as an anti-apoptotic factor in Mtb ([44,45]). The discovery of *nuoG* and NDH-1 as anti-apoptosis factors encoded by specific Mtb genes suggests new strategies for improving currently used and novel tuberculosis vaccines, and could also provide targets for development of antimicrobial drugs for treatment of persistent disease.

## Materials and Methods

### Bacteria and culture conditions.

M. smegmatis (mc^2^155) has been previously described [35], and M. kansasii strain Hauduroy ( ATCC 12478) and Mtb strain H37Rv (ATCC 25618) were obtained from the American Type Culture Collection (http://www.atcc.org/). M. bovis BCG Pasteur strain was obtained from the Trudeau Culture Collection (Saranac Lake, New York, United States). GFP-expressing BCG and M. smegmatis were generated by subcloning the enhanced GFP gene (Clontech, http://www.clontech.com/) into the mycobacterial episomal expression vector pMV261. The resulting plasmid (pYU921) was transfected into competent cells by electroporation as previously described [35]. M. smegmatis was cultured in LB broth with 0.5% glycerol, 0.5% dextrose, and 0.05% TWEEN-80. Mtb H37Rv and M. kansasii were grown in 7H9 broth with 0.5% glycerol, 0.5% dextrose, 0.05% TWEEN-80, and 10% OADC enrichment (DIFCO, http://www.bd.com/ds/). For selective media, 50 μg/ml hygromycin or 40 μg/ml kanamycin were added.

### Cell culture conditions and infection.

Human myelomonocytic cell line THP-1 (ATCC TIB-202) was cultured and differentiated using phorbol myristate acetate (PMA) (Sigma, http://www.sigmaaldrich.com/) as described [46]. Bone marrow macrophages were derived from the femur and tibia of BALB/c mice as described [46]. Bacteria were grown to an OD_600_ ranging from 0.5 to 0.8, sonicated twice for 20 s using a cup horn sonicator, and allowed to settle for 10 min. The infection was carried out at a multiplicity of infection (MOI) of 10:1 (10 bacilli to 1 cell) for 4 h in triplicate wells, after which extracellular bacteria were removed by four washes with phosphate buffered saline (PBS). The cells were incubated in DMEM (Invitrogen, http://www.invitrogen.com/) with 20% human serum (Sigma) and 100 μg/ml gentamicin (Invitrogen), and an apoptosis assay was performed after the indicated periods of culture.

### Apoptosis assay.

The TUNEL assay was performed to reveal apoptosis-induced DNA fragmentation in either tissue culture cells or lung sections of infected mice using the In Situ Cell Death Detection Kit, Fluorescein (for cultured cells) or In Situ Cell Death Detection Kit, POD (for lung sections) (Roche Applied Science, http://www.roche-applied-science.com/). The assay was carried out as described by the manufacturer and the percentage of stained cells was analyzed using flow cytometry for cultured cells or quantification via light microscopy for the animal tissue sections.

### Construction of Mtb genomic DNA library.

The strategy for generation of the Mtb genomic library in cosmid vector pYUB415 has been previously described [47]. Briefly, Mtb (strain Erdman) genomic DNA was purified and partially digested with Sau3A. DNA fragments of about 40 kbp were selected by agarose gel purification and ligated into arms of cosmid vector pYUB415 digested with BamH1 as previously described [47]. DNA was packaged in vitro with Gigapack XL (Stratagene, http://www.stratagene.com/) and Escherichia coli were transduced and selected on LB plates containing 100 μg/ml ampicillin. Over 10^5^ independent clones were pooled, and DNA for transformation was obtained using standard alkaline lysis method.

### Transformation and gain-of-function screen.

Transformations were performed by electroporation of competent mycobacteria as described [35]. For the initial screen, M. smegmatis was transformed with the genomic DNA cosmid library described above, and 312 cosmid clones were picked and grown in liquid medium containing 50 μg/ml hygromycin. Assuming random distribution of Mtb sequences among the cosmid transformants and an average insert size of about 40 kbp, the 312 cosmid clones represented 3-fold coverage of the entire Mtb genome. After three successive screens using bright field microscopy or flow cytometry to assess levels of cell death, 12 clones were selected for quantitative assessment using TUNEL staining followed by flow cytometry. This identified three clones of greatest interest (M24, J21, and I16), and their cosmid DNA was purified and screened by restriction digest (not shown). This revealed that the inserts of M24 and I16 were identical, but different from the insert of J21. For cosmid J21, the 5′ and 3′ ends of the insert DNA were sequenced and aligned with the published genomic Mtb DNA sequence (Figure S2), and subsequently the whole insert was sequenced to confirm that is corresponded to the sequence published data.

### Specialized transduction for construction of deletion mutants in Mtb*.*

Specific genes of Mtb were disrupted using specialized transduction as described [37]. To create the *nuoG::hyg-null* allele, the hygromycin resistance cassette was introduced between the first 4 bp of the *nuoG* 5′ end and the last 163 bp of the 3′ end of the open reading frame. The successful deletion of the gene was demonstrated by Southern blotting as described previously (Figure S3). For complementation of the Δ*nuoG* mutation, *nuoG* was amplified by PCR and cloned behind the *hsp60* constitutive promoter into the plasmid pMV361, which allows integration of a single copy into the genome of Mtb [38].

### Animal studies.

BALB/c or SCID/Ncr (BALB/c background) mice (4- to 6-wk-old females) were infected intravenously through the lateral tail vein with 1 × 10^6^ bacteria. For survival studies, groups of ten mice were infected, and after 24 h, three mice per group were sacrificed to determine the bacterial load in the organs. In order to follow the bacterial growth, an additional three mice per time point were infected. The organs (lung, spleen, liver) were homogenized separately in PBS/0.05% TWEEN-80, and colonies were enumerated on 7H10 plates grown at 37 °C for 3–4 wk. For histopathology, tissues were fixed in 10% buffered formalin and embedded in paraffin; 4-μm sections were stained with hematoxylin and eosin. TUNEL staining was performed on the paraffin-embedded tissue sections using the In Situ Cell Death Detection Kit, POD (Roche Applied Science) per the manufacturer's protocol. Quantification was performed on coded specimens by a blinded observer by counting the number of apoptotic nuclei per ∼200 total nuclei in eight separate areas of two lung sections for each of the three mice per group. All animals were maintained in accordance with protocols approved by the Albert Einstein College of Medicine and University of Maryland Institutional Animal Care and Use Committees.

## Supporting Information

Figure S1 Representative Lung Sections of Infected and Uninfected SCID Mice (27.6 MB PPT)

Figure S2 Insert of Cosmid J21 (43 KB PPT)

Figure S3 Creation of *nuoG* Null Mutant in M. tuberculosis
(90 KB PPT)

Figure S4 Identification of *nuoG* as a Mycobacterial Virulence Gene (95 KB PPT)

Figure S5 TUNEL Staining of Lung Sections of Infected SCID Mice (811 KB PPT)

Figure S6 TUNEL Staining of Lung Sections of Infected BALB/c Mice (528 KB PPT)

Figure S7 A nuoG-phoA Fusion Protein Was Not Secreted (192 KB PPT)

Figure S8 nuoG Deletion Did Not Lead to a Major Change in Mtb Proteome (7.8 MB PPT)

Figure S9 nuoG Protein Sequence Alignment (159 KB PPT)

Figure S10 Similar Bacterial Burden in SCID Mice Infected with Mtb, Mutant, and Complemented Mutant Strain after 24 h (95 KB PPT)

### Accession Numbers

The GenBank (http://www.ncbi.nlm.nih.gov/Genbank/index.html) accession numbers for the sequences of the nuoG proteins from the following mycobacteria are *M.bovis* AF2122/97 (CAD95267), M. bovis BCG-Pasteur (YP979258), M. kansasii Hauduroy (EF607211), M. smegmatis mc^2^155 (YP886418), M. tuberculosis CDC1551 (AAK47578), and M. tuberculosis H37Rv (CAB06288).

## Abbreviations

BCG: *M. bovis* Bacille Calmette-Guérin
CFU: colony-forming unit
Mtb: *Mycobacterium tuberculosis*
NDH-1: type I NADH dehydrogenase complex
